# Fine-Scale Exposure to Allergenic Pollen in the Urban Environment: Evaluation of Land Use Regression Approach

**DOI:** 10.1289/ehp.1509761

**Published:** 2015-10-09

**Authors:** Jan Hjort, Timo T. Hugg, Harri Antikainen, Jarmo Rusanen, Mikhail Sofiev, Jaakko Kukkonen, Maritta S. Jaakkola, Jouni J.K. Jaakkola

**Affiliations:** 1 Geography Research Unit, and; 2 Center for Environmental and Respiratory Health Research, University of Oulu, Oulu, Finland; 3 Finnish Meteorological Institute, Helsinki, Finland

## Abstract

**Background:**

Despite the recent developments in physically and chemically based analysis of atmospheric particles, no models exist for resolving the spatial variability of pollen concentration at urban scale.

**Objectives:**

We developed a land use regression (LUR) approach for predicting spatial fine-scale allergenic pollen concentrations in the Helsinki metropolitan area, Finland, and evaluated the performance of the models against available empirical data.

**Methods:**

We used grass pollen data monitored at 16 sites in an urban area during the peak pollen season and geospatial environmental data. The main statistical method was generalized linear model (GLM).

**Results:**

GLM-based LURs explained 79% of the spatial variation in the grass pollen data based on all samples, and 47% of the variation when samples from two sites with very high concentrations were excluded. In model evaluation, prediction errors ranged from 6% to 26% of the observed range of grass pollen concentrations. Our findings support the use of geospatial data–based statistical models to predict the spatial variation of allergenic grass pollen concentrations at intra-urban scales. A remote sensing–based vegetation index was the strongest predictor of pollen concentrations for exposure assessments at local scales.

**Conclusions:**

The LUR approach provides new opportunities to estimate the relations between environmental determinants and allergenic pollen concentration in human-modified environments at fine spatial scales. This approach could potentially be applied to estimate retrospectively pollen concentrations to be used for long-term exposure assessments.

**Citation:**

Hjort J, Hugg TT, Antikainen H, Rusanen J, Sofiev M, Kukkonen J, Jaakkola MS, Jaakkola JJ. 2016. Fine-scale exposure to allergenic pollen in the urban environment: evaluation of land use regression approach. Environ Health Perspect 124:619–626; http://dx.doi.org/10.1289/ehp.1509761

## Introduction

Asthma is globally the most common chronic disease in children, and it affects approximately 7.7% of the working-age population in the United States (Henneberger et al. 2011) and 3–9% of adults in Finland and in other parts of Europe (Boutin-Forzano et al. 2007; Jaakkola et al. 2002; Pallasaho et al. 2011). Allergies are even more common, with a prevalence of 20–40% among children (Aït-Khaled et al. 2009; Asher et al. 2006). Emerging climate change will influence temperature, precipitation, and the spatial distribution of pollen species with strong allergenic properties, and these changes may have profound effects on both the etiology of asthma and allergies and the occurrence of symptoms among subjects with these diseases (e.g., Beggs and Bambrick 2005; Cecchi et al. 2010; D’Amato et al. 2014; Gilmour et al. 2006).

Grass (*Poaceae*) pollen is the most widespread group of pollen allergens worldwide, and is the most frequent cause of pollen allergy in Europe and one of the most common causes in the United States (D’Amato et al. 2007; White and Bernstein 2003). In general, most individuals suffering from allergic rhinitis experience seasonal pollen-related symptoms (Blomme et al. 2013). Pollen allergy has been identified in 80–90% of children with asthma and 40–50% of adults with asthma (Taylor et al. 2007). The importance of pollen is highlighted in urban environments, where the prevalence of allergy has been estimated to be higher than in rural environments (e.g., Majkowska-Wojciechowska et al. 2007; Priftis et al. 2007).

Conditions that promote plant growth and reproduction, such as higher temperatures and carbon dioxide concentrations (Singer et al. 2005; Ziska et al. 2003), have been shown to increase pollen production and promote an earlier start of pollen season in urban areas compared with surrounding rural areas (Ziska et al. 2003). Importantly, there is evidence that the allergenic potential of polluted (urban) pollen is stronger than that of nonpolluted pollen grains (Aina et al. 2010; Majd et al. 2004). Thus, relatively more favorable vegetation growth conditions, higher pollen counts, earlier start of pollen season, and greater allergenic potency may have a pronounced effect on the occurrence and the severity of allergic symptoms and on the risk of asthma and allergies in urban environments (Beggs 2004; Gilmour et al. 2006; Ziska et al. 2003). The global trend of urbanization and formation of mega-cities will increase the human exposure to mixtures of chemical and biological pollutants [World Health Organization (WHO) 2013].

Previous epidemiologic studies have predominantly assessed exposure on the basis of pollen data from only one or few monitoring stations (Caillaud et al. 2014; Delfino et al. 2002). An additional limitation in several studies is that pollen data have been based on roof-level measurements (e.g., at the heights of 10–30 m) and thus do not reflect properly the most common exposure at breathing level (e.g., 1.5 m). Roof-level samplers collect particles from a larger geographic area, reflecting mainly the influences of regional sources of pollen grains (O’Rourke and Lebowitz 1984). Thus, roof-level data do not address intra-urban spatial heterogeneity of pollen concentrations, and they are less accurate for exposure assessment when studying potential human health effects (Peel at al. 2013). Consequently, there is an urgent need for studies that can quantify the relations between environmental determinants and allergenic pollen concentrations across urban gradient at local (≤ 100–300 m) scales (Haberle et al. 2014; WHO 2013).

In recent years, there has been significant progress in the modeling of transportation and concentration of atmospheric pollen at large spatial scales (Kukkonen et al. 2012; Skjøth et al. 2013; Sofiev and Bergmann 2012; Sofiev et al. 2013). However, even the most sophisticated current methods cannot estimate the concentrations of pollens at fine spatial scale. In this context, a land use regression (LUR) approach based on readily available geographic information system (GIS) data could provide a cost-efficient and reasonably accurate method for predicting the variability of pollen concentrations at fine spatial scales (Beelen et al. 2013; Richardson et al. 2013).

The overall objective of this study was to improve and evaluate the LUR methodology for the prediction of spatial variability of allergenic pollen concentrations in the urban area. We focused on grasses, as one of the most important allergenic plant groups. More precisely, we addressed the following specific objectives: *a*) to model intra-urban spatial variation of grass pollen concentrations and *b*) to determine the best environmental predictors of intra-urban variation in grass pollen concentrations.

## Methods

### Study Area

**Figure 1:**
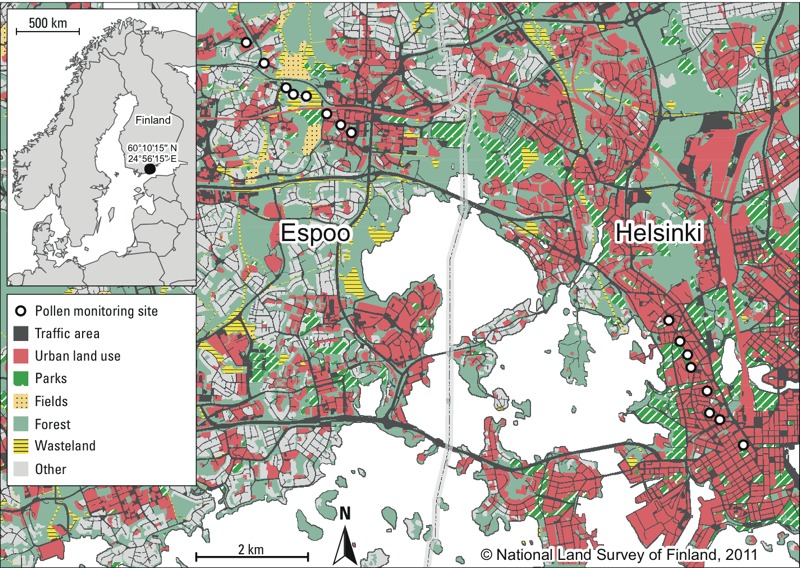
Land use of the study area and the location of the monitoring sites for grass pollen [ESRI Data & Maps 9.3; ArcWorld Supplement; National Land Survey of Finland (http://www.maanmittauslaitos.fi/en/kartat)]. Urban land use refers to high-density (e.g., block of flats, commercial and industrial) land use types, parks to managed grasslands, fields to cultivated fields, and wasteland to unmanaged grasslands.

The study was conducted in the Helsinki metropolitan area (1.1 million inhabitants), southern Finland (60°10′15″N, 24°56′15″E) (Figure 1). The study area includes both constructed urban environments with a limited amount of vegetation and natural environments covered by diverse vegetation. The study area has the characteristics of both maritime and continental climates. The mean annual temperature is 5.9°C and the mean annual precipitation is 655 mm (1981–2010; Pirinen et al. 2012). The area belongs to the temperate coniferous–mixed forest zone. Grasses are typical pioneer plant groups (i.e., those that are the first to colonize newly exposed land surfaces) in the ground layer of the area.

### Data Collection

*Grass pollen data.* Two sampling lines, each with a length of 3 km, were placed within the cities of Helsinki and Espoo (Figure 1). The selected sampling sites differed from each other with respect to land use and vegetation type. These two categories of urban environments (urban environment in central Helsinki, and residential suburban area in Espoo) were selected for evaluation because grass pollen exposures in these areas may be representative of the exposures experienced by many urban populations. Altogether, pollen grains were monitored at 16 different sites (1.5 m above ground surface) during the grass pollen season in 2013. The sampling was conducted daily (except on rainy days) from 27 June through 21 July 2013, during the peak grass pollen season. See Supplemental Material, Table S1, for a summary of weather conditions during the sampling period.

Rotorod-type samplers were used for pollen monitoring (Rantio-Lehtimäki et al. 1992). To minimize problems with oversampling (Sterling and Lewis 1998), samples were collected at each site for only 30 min each morning (between 0800 and 1130 hours) and each afternoon (between 1300 and 1630 hours). On each day the specific sampling time differed among the individual sites, and the specific sampling times at each site differed from day to day. In addition, sampling days alternated between the eight Helsinki sites and the eight Espoo sites (Figure 1). For additional details, see Supplemental Material, “Sampling of grass pollen data.” Pollen measurements were converted into volumetric equivalents expressed as the concentration of pollen grains per cubic meter of air sampled. The grass pollen data were subdivided into three subsets that each represented the average concentration of pollens at the breathing zone (1.5 m) at each one of the 16 sampling sites during a 2-week period. The first was used to calibrate the LUR model, and included data collected June 27–July 9 during the afternoon sampling period (1300–1630 hours). The second and third were used for independent evaluation of the model, with the second collected 27 June–9 July during the morning sampling period (0800–1130 hours), and the third collected 10–21 July during the morning and afternoon sampling period. In the calibration data set, average pollen concentrations for 2 of the 16 sites were very high (120.2 and 1758.0 grains/m^3^) compared with average concentrations for the other 14 sites (1.4–23.9 grains/m^3^). Therefore, in addition to analyzing the data from all 16 sites (hereafter referred to as the *n* = 16 data set), we performed a second set of analyses after excluding the data from the 2 sites with very high concentrations (hereafter referred to as the *n* = 14 data set) in order to assess the robustness of our model with regard to potential outliers.

*Environmental determinants.* Altogether, eight GIS-based geospatial environmental determinants (Table 1) were computed at 25-, 50-, 100-, 300-, 500-, and 1,000-m buffer sizes (Lovasi et al. 2013). The range of buffer sizes was selected based on the knowledge of dispersal of grass pollens (e.g., Skjøth et al. 2013) and the properties of geospatial data sets (e.g., accuracy and resolution). The applied determinants included two remote sensing–based indices [tasseled cap transformation (TC) brightness and TC greenness (Lillesand et al. 2004)], four land use determinants (wasteland, park, field, and urban land use) compiled from the 2010 SLICES land use classification [National Land Survey of Finland (http://www.maanmittauslaitos.fi/en/kartat)] and two land use/cover variables (deciduous forest and mixed forest) computed using the 2006 CORINE land cover database (Finnish Environmental Institute 2009). The values of the environmental determinants were computed using ArcGIS 10.2 (ESRI) with buffers at various sizes.

**Table 1:** Environmental determinants computed to explore the intra-urban variation of grass pollen concentrations.

Environmental variable	Unit	Description	Source
TC brightness	Index	Land use/cover with high albedo (overall brightness of the image)	Landsat TM5
TC greenness	Index	Amount of photosynthetically active green vegetation	Landsat TM5
Wasteland	m^2^	Unmanaged grasslands (e.g., meadows and power lines)	SLICES land use classification
Park	m^2^	Managed grasslands (e.g., urban parks and sports fields)	SLICES land use classification
Field	m^2^	Cultivated fields (e.g., wheat and barley fields)	SLICES land use classification
Deciduous forest	m^2^	Broadleaf trees (e.g., birch, alder, and maple)	CORINE land cover database
Mixed forest	m^2^	Broadleaf trees and conifers (pine and spruce)	CORINE land cover database
Urban land use	m^2^	Urban land use classes (e.g., high density residential, commercial, industrial, and traffic areas)	SLICES land use classification
Abbreviations: TC, tasseled cap; TM5, Thematic Mapper 5. The Landsat data were from 2010 (http://earthexplorer.usgs.gov/), SLICES data were from 2010 [National Land Survey of Finland (http://www.maanmittauslaitos.fi/en/kartat)], and CORINE from 2006 (Finnish Environmental Institute 2009).			

**Figure 2:**
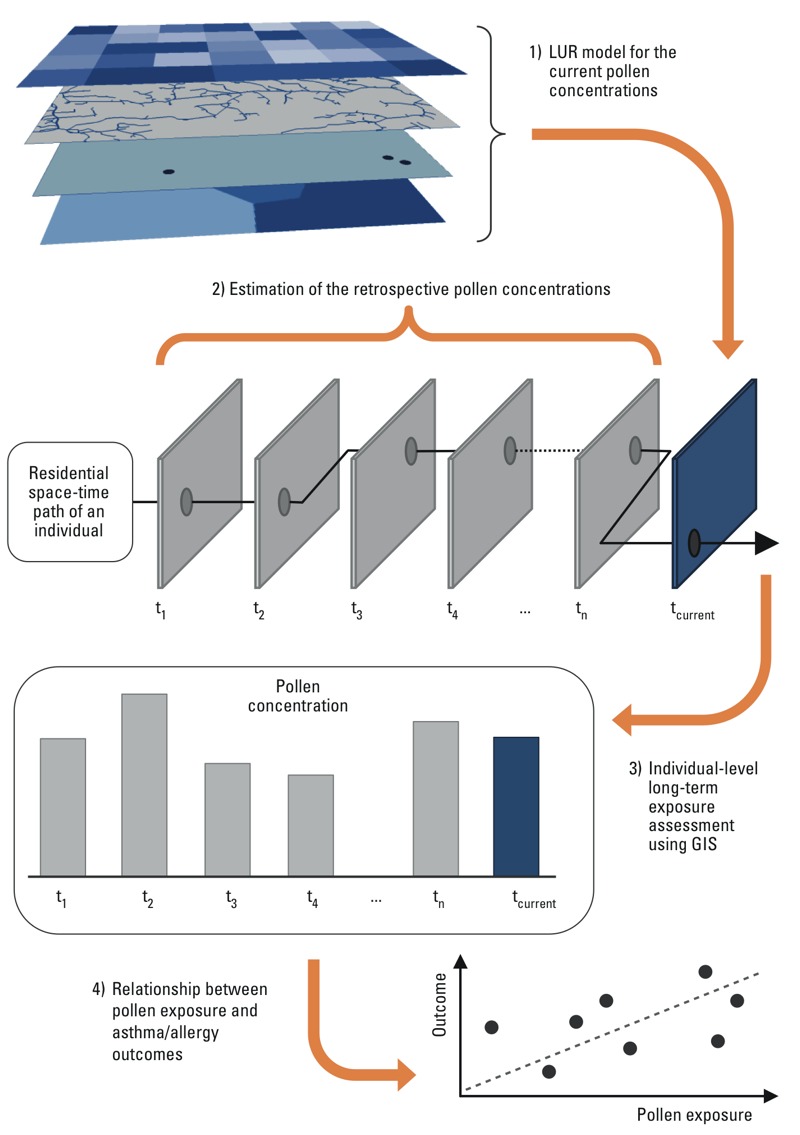
Application of the land use regression (LUR) outcomes (e.g., average seasonal concentration of pollen) in long-term and lifetime exposure assessments. First, the LUR approach is used to produce a raster model of pollen concentrations in the current environmental conditions (t_current_). Second, the retrospective pollen concentrations (t_1_…t_n_) are predicted using data describing the past environmental conditions (e.g., historical land use and remote sensing data) (Gulliver et al. 2013). In the estimation of retrospective concentrations, long-term (permanent) pollen collectors are used. Third, geographic information system (GIS) tools are used to compute individual (e.g., cohort members) exposure based on residential history data at applicable spatial resolution (Richardson et al. 2013). Fourth, the results of the previous step are used in allergy and asthma explorations.

The source of the remote sensing data was selected based on the following criteria: The images should be freely available, they should have global coverage with high spatial resolution (10–50 m), and the data should be available for several decades (to facilitate linking this data to long-term epidemiologic cohort data; e.g., Lovasi et al. 2013) (Figure 2). Consequently, the remote sensing–based indices—TC brightness and TC greenness (Lillesand et al. 2004)—were computed from orthorectified Landsat TM5 satellite image (considering the acquisition day and cloud cover the most suitable image was from 14 July 2010) using Erdas IMAGINE. We selected the TC greenness variable instead of the more commonly used Normalized Difference Vegetation Index (NDVI) (e.g., Skjøth et al. 2013) based on a preliminary analysis that indicated that the TC greenness variable was more highly correlated with grass pollen concentrations [the highest Spearman’s rank order correlation coefficients (*r*_s_) = 0.792, *p* < 0.001] than the NDVI (the highest *r*_s_ = 0.788, *p* < 0.001).

### Statistical Analysis

Before the multivariate statistical analyses, the distributions of the grass variables were normalized using logarithmic (log_10_) transformation (normality was statistically confirmed applying the Kolmogorov–Smirnov test) (Sokal and Rohlf 1995). The statistical analyses were conducted in three steps to explore the explanation and prediction ability of the selected environmental determinants at various scales. The main statistical methods applied were Spearman’s rank correlation analysis (Sokal and Rohlf 1995), hierarchical partitioning (HP; Chevan and Sutherland 1991), and generalized linear modeling (GLM; McCullagh and Nelder 1989). First, *r*_s_ between grass pollen concentrations and environmental determinants were calculated to select the optimal buffer sizes. The selection of the optimal buffer size was based on the highest correlation coefficients, also requiring an expected sign (e.g., a positive sign for variables describing potential environments for grasses). The correlations were calculated with IBM SPSS Statistics 19 software. Second, to explore the potential effects of multicollinearity in statistical analyses (i.e., intercorrelation among environmental determinants), we applied HP (Chevan and Sutherland 1991) as described in detail in Supplemental Material, “Hierarchical partitioning.” Third, GLM was employed to model the observed grass pollen concentration differences in the study area. The calibration of the GLM was performed using the standard *glm* function in R (R Core Team 2015). The GLM optimization was based on the forward-selection approach and Akaike’s Information Criterion (AIC) (Burnham and Anderson 2004). We evaluated the calibrated model on the basis of the model fit to the data, normality of the residuals (applying the Kolmogorov–Smirnov test), homoscedasticity, spatial independency (applying the Moran’s *I*), and leverage (Beelen et al. 2013; Sokal and Rohlf 1995).

The predictive ability of the final GLMs was assessed using leave-one-out cross-validation (CV) of models based on the calibration data set. In addition, we performed an external validation by comparing calibration model predictions with the two evaluation data sets based on samples collected 27 June–9 July during the morning, and samples collected 10–21 July during the morning and afternoon, respectively. In addition, we visually compared spatial variation in the predicted concentrations with land use and land cover characteristics across the Helsinki metropolitan area.

## Results

### Optimization of the Buffer Sizes

Environmental variables with the highest Spearman’s rank order correlations with grass pollen concentrations based on the full (*n* = 16) data set were TC greenness (optimum buffer size = 50 m, *r*_s_ = 0.79, *p* < 0.001), wasteland (300 m, *r*_s_ = 0.73, *p* < 0.001), urban land use (300 m, *r*_s_ = –0.72, *p* < 0.01), field (1,000 m, *r*_s_ = 0.67, *p* < 0.01), and deciduous forest (300 m, *r*_s_ = 0.63, *p* < 0.01) (see Supplemental Material, Table S2). For the reduced (*n* = 14) data set, grass pollen concentrations correlated highest with TC greenness (50 m, *r*_s_ = 0.69, *p* < 0.01), urban land use (1,000 m, *r*_s_ = –0.62, *p* < 0.05), wasteland (500, *r*_s_ = 0.62, *p* < 0.05), and mixed forest (50 m, *r*_s_ = 0.62, *p* < 0.05) (see Supplemental Material, Table S3). Thus, the correlation patterns were rather similar for the two data sets, although correlations were weaker for the *n* = 14 data set than for the full data set.

### Hierarchical Partitioning

The results of the HP analysis—the independent contribution of the environmental determinants at the optimum buffer size—are presented in Supplemental Material, Figure S1. The contributions of the TC greenness and urban land use variables were > 15% in both data sets (range, 17–30%). Moreover, the wasteland variable contributed 22% in the *n* = 16 data set and the mixed forest variable 21% in the *n* = 14 data set. Because the sample sizes were small, only the TC greenness variable was statistically significant (*p* < 0.001) in the larger data set, and none of the determinants were significant (*p* > 0.05) in the smaller data set.

### Calibration and Evaluation of Generalized Linear Models

The final GLMs for the *n* = 16 and *n* = 14 data sets both included the TC greenness variable, and the *n* = 16 model also included the wasteland variable. The two GLMs explained 79% and 47% of the variation in the grass pollen data, respectively (D^2^ values, Table 2). Based on the exploration of residuals, the assumptions of normal errors and independency were not statistically violated. The larger data set included one potential outlier (Cook’s distance > 1), but when an alternative GLM was calibrated without the outlier observation, the normality and homoscedasticity of the residuals was reduced, and a new potential outlier was identified (data not shown).

**Table 2:** Results of the final generalized linear models (GLMs).

Model calibration	All samples (*n *= 16)	Extreme values off (*n *= 14)
Final GLM (α + βx_i_)	1.287 + 0.032 (greenness) + 7.8^–6 ^(wasteland)	1.179 + 0.024 (greenness)
AIC	18.2	8.0
D^2^	0.785	0.466
Correlation (fitted – obs)	*r* = 0.89 (*p *< 0.001); *r*_s_ = 0.79 (*p *< 0.001)	*r* = 0.68 (*p *= 0.007); *r*_s_ = 0.69 (*p *= 0.006)
Residuals
Normality (K-S test)	Normally distributed (*p *= 0.829)	Normally distributed (*p *= 0.826)
Homoscedasticity	Acceptable	Acceptable
Spatial autocorrelation	No autocorrelation (Moran’s *I* *p *> 0.05)	No autocorrelation (Moran’s *I* *p *> 0.05)
Cook’s distance	One observation > 1	All observations < 0.5
Abbreviations: α, intercept; β, regression coefficient; x_i_, environmental variable; AIC, Akaike’s Information Criterion; D^2^, explained deviance (comparable to explained variance in least-square regression); fitted, fitted values; greenness, greenness of tasseled cap transformation; K-S test, Kolmogorov–Smirnov test; obs, observed values; *r*, Pearson’s correlation coefficient; *r*_s_, Spearman’s rank order correlation coefficient; wasteland, unmanaged grassland land use classes.		

**Figure 3:**
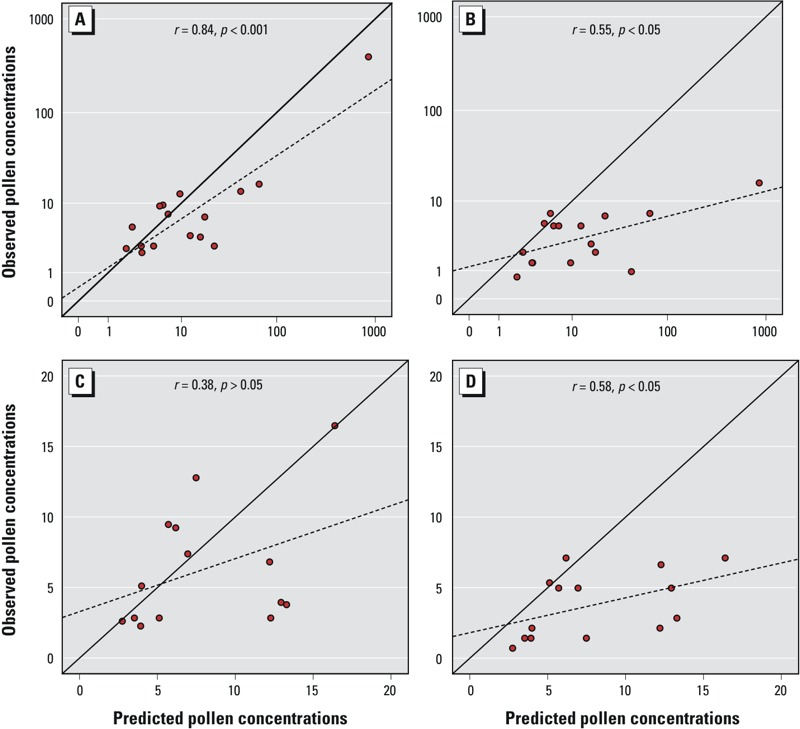
Scatter plots of the observed (i.e., measured) and predicted grass pollen concentrations (grains/m^3^) for the larger (*n *= 16) (*A,B*) and smaller (*n *= 14) (*C,D*) data sets. In the evaluation setting, the observed concentrations were measured during morning (0800–1130 hours, 27 June–9 July 2013) (*A*,*C*) and another period (0800–1130 and 1300–1630 hours, 10–21 July 2013) (*B*,*D*). The solid line shows the optimum fit (intercept = 0, slope = 1) and the dashed line the fit to the data. Pearson’s correlation coefficients (*r*) are also shown.

Based on the CV results, the prediction errors were 412.9 grains/m^3^ and 5.9 grains/m^3^ for the *n* = 16 and *n* = 14 data sets, respectively. However, the mean prediction errors were 6.4% (*n* = 16) and 19.5% (*n* = 14) and root-mean-square errors were 23.5% (*n* = 16) and 26.4% (*n* = 14) of the observed range of grass pollen concentrations. The calibrated model for the full data set predicted the measured concentrations of samples collected during the morning (instead of the afternoon) on the same days better than it predicted measured concentrations during the second observation period (10–21 July) (Pearson’s *r* = 0.84, *p* < 0.001 and 0.55, *p* < 0.05, respectively) (Figure 3A,B). In contrast, predictions based on the *n* = 14 data set were slightly better for the second observation period than for the morning samples (*r* = 0.58, *p* < 0.05 and 0.38, *p* > 0.05, respectively) (Figure 3C,D).

**Figure 4:**
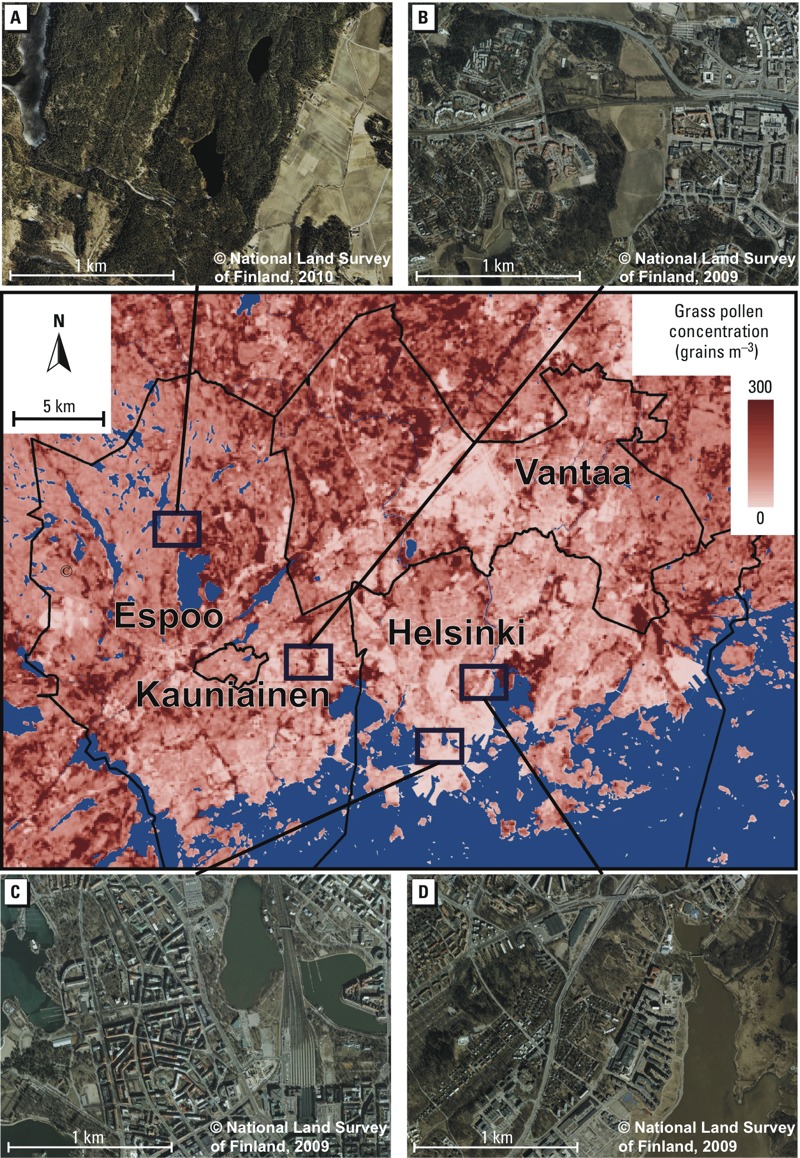
A smoothed prediction map of the grass pollen concentration (grains/m^3^; afternoon situation during 27 June–9 July 2013) at a 100-m resolution and aerial photographs (*A–D*) (resolution = 50 cm) representing different environmental conditions within the Helsinki metropolitan area, Finland. The predictions were computed using the final generalized linear model and *n* = 14 data set. To consider the potential statistical problems related to outlier observations in the *n* = 16 data set (see the “Methods” and “Results” sections) the smaller data set was used in the prediction (i.e., in the extrapolation of the model beyond the environmental conditions of the calibration area) [National Land Survey of Finland (http://www.maanmittauslaitos.fi/en/kartat)].

The overall view of the predicted concentrations of grass pollen follows well the land use patterns in the Helsinki metropolitan area (Figure 4). For example, the major source areas of pollens (e.g., grasslands and open semi-natural land use types; Figure 4A,D) and areas of intensive land use (e.g., built-up and traffic areas; Figure 4B–D) can be identified. Moreover, that the most densely vegetated environments (i.e., forest; Figure 4A) are not the areas with the highest concentrations of grass pollens. This result is to be expected, because grasses do not flourish in forests with closed canopy. On the contrary, the models appear to predict too high grass pollen concentrations for some of the cultivated fields (Figure 4A,B) and managed grass areas (e.g., golf courses). However, the models predict realistically rather low concentrations for extensively managed grass areas—for example, around the airport runways in the middle part of the city of Vantaa (Figure 4).

## Discussion

Atmospheric concentration of allergenic pollen is an important public health indicator, the potential of which has not been fully utilized (Ring et al. 2014; WHO 2013). Although the role of pollen in the onset of allergic disease is not well established, exposure to pollen generates symptoms among asthmatics and allergic individuals (Gilmour et al. 2006; Zeldin et al. 2006). Thus, improved prediction of the variability of allergenic pollen concentrations at local scales would be valuable when studying the role of pollen exposure in the development of allergic diseases and sensitization, as well as exacerbations of symptoms among subjects with asthma and allergies. This would also pave the way for improved predictions of the future health impact of climate change through changes in the generation and distribution of pollen.

The LUR models developed in this study are designed for predicting the variability of the breathing-zone level concentrations of allergenic grass pollens in urban environment with very high resolution using multi-source geospatial data. First, this is of importance because physically based dispersion models are not, at least yet, applicable in the exploration of fine-scale spatial variation of pollen concentrations (Sofiev and Bergmann 2012; Sofiev et al. 2013). The main application of the suggested methodology is to assess longer-term (from weekly to seasonal) exposures to pollens and not short-term, high-dose exposures (Figure 2). Second, the measured grass pollen concentrations presented significant intra-urban variation. Consequently, it is necessary to consider also local-scale sources in addition to regional-scale sources or long-distance atmospheric transport when assessing population exposure to grass pollens in urban environments.

### Implications for the Assessment of Exposure and Health Effects

Associations between pollen concentrations measured by regional pollen monitoring stations and allergic and asthmatic symptoms and lung function variation have been reported (Caillaud et al. 2014; Delfino et al. 2002). However, the role of long-term exposure to allergenic pollen in the development of asthma and other allergic diseases has not been studied, partly because of the lack of appropriate methods for exposure assessment.

Our findings suggest that allergenic grass pollen concentrations can be estimated with reasonable accuracy using geospatial data variables. Because similar data have been collected for several decades, it may be possible to adapt our method to perform retrospective lifetime exposure assessment (Gulliver et al. 2013) for cohort members based on historical land use data, pollen data, and individual residential history data (Richardson et al. 2013) (Figure 2). In this example, the local variations in pollen concentration are predicted using past environmental conditions (e.g., Landsat satellite images that are available for more than 3 decades), and the regional level of concentration is determined by a permanent, long-term pollen collector located in the study area (e.g., daily pollen observations from the Helsinki metropolitan area have been available since the mid-1970). The presented modeling approach could also be applied in cross-sectional studies comparing the prevalence of asthma-related and allergic symptoms according to residential area or time-activity pattern.

One notable issue was the moderate prediction bias when the models were extrapolated to other periods (i.e., under-prediction of grass pollen concentrations when pollen production of the extrapolation period was lower than in the calibration period). However, this potential bias can be taken into account in retrospective predictions, if the overall concentration differences between different periods are known. The long-term pollen concentration measurements from regional monitoring stations can be used for this purpose.

### Data and Methodological Issues

Our findings reinforce the need to identify optimal buffer sizes for each environmental determinant, because appropriate buffers may vary substantially. In addition, our results support the hypotheses that there are large intra-urban variations in grass pollen concentrations, and these heterogeneities are associated with local-scale variations in land use (Skjøth et al. 2013). The remote sensing–based TC greenness variable outperformed land use and land cover variables in this study. In general, remote sensing–based indices have several advantages over the traditional land use variables. Remote sensing data can be acquired over extensive areas and from numerous sources at various spatial, temporal, spectral and radiometric resolutions (Lillesand et al. 2004). More important, remote sensing–based variables can be computed to describe the environmental conditions continuously (i.e., each pixel has a continuous numerical value).

In contrast, the land use variables are dichotomized (i.e., the class is present or not) (Katz and Carey 2014). This property impedes the use of distinct land use/cover classes in predictive modeling settings, because the model will predict equal concentrations in areas without variation in the explanatory variable(s). For example, the wasteland variable describing unmanaged grasslands was, in theory, an ideal variable reflecting the sources of grass pollens. However, the amount of this land use type was low in the study area (Figure 1).

Some data-related and statistical issues should be considered. First, the number of observation sites (*n* = 16) was limited, which caused challenges in model calibration and evaluation. Robust statistical models usually require dozens of observations (e.g., Hjort et al. 2011; Beelen et al. 2013), and hundreds of sample sites may be optimal in analyzing ecological response variables (Stockwell and Peterson 2002). The use of passive aerosol samplers would make it feasible to acquire data from more sites and thus improve the assessment and prediction of spatial variation in pollen concentrations in intra-urban areas (e.g., Hofmann et al. 2014).

Second, variables describing meteorological conditions were not included into the set of environmental determinants. The primary goal of our model was to predict the spatial variability of pollen concentrations within an urban area: Additional data and a longer sampling period would be required to accurately predict the absolute values of the pollen concentrations. The weather conditions were partially taken into account in the sampling of pollen data (no sampling during rainfall, only daytime sampling) and during the preparation of calibration and evaluation data sets (see Supplemental Material, Table S1). Implicitly, the regular effects of meteorological factors in different land use classes are accounted for during the calibration step. We assumed that all other effects of meteorology would be consistent across the study area, which would make them irrelevant for predicting spatial variation. Moreover, we used data averaged over 2-week periods (27 June–9 July and 10–21 July, respectively) to reduce the impact of short-term temporal variability.

Third, in addition to the sample size–related problems, regression-based statistical methods include several data-based assumptions (Sokal and Rohlf 1995). For example, assumptions of normality, homoscedasticity, and independence of errors are often violated in analyzing complex responses. In this study, we aimed to minimize problems related to regression analysis in compilation and computation of study material [e.g., comprehensive explorative data analysis (Tukey 1977) before multivariate analyses; data not shown] and by using generalization of the least-square linear regression method (i.e., GLM). Moreover, we used widely accepted model calibration and evaluation procedures (e.g., Beelen et al. 2013) and the HP method for considering potential multicollinearity problems in multivariate analysis (Chevan and Sutherland 1991).

For future studies focusing on spatial prediction of pollen concentrations for exposure assessments, we recommend the following steps (Elith and Leathwick 2009; Beelen et al. 2013): *a*) establishment of a conceptual model that is based on literature and empirical findings, *b*) compilation of environmental data from different sources and at various scales (remote sensing–based at finer and land use at coarser scales), *c*) comprehensive statistical and graphical exploration of both response and environmental variable data, and *d*) substantial evaluation of the generated model. The evaluation should include the assessment of the realism of fitted explanatory variables (e.g., expected signs for regression coefficients), the model’s fit to data, characteristics of residuals, predictive performance on independent test data, and visual/graphical exploration of the predictions.

## Conclusions

In this study, we have elaborated the relationship between environmental determinants and atmospheric allergenic pollen concentrations in an urban area. Previous studies have not explored the possibility of predicting the intra-urban variation of grass pollen concentrations, using geospatial data and statistical methods. A novelty of the present work is a comprehensive set of pollen measurements in urban environments, which enables the spatial modeling of urban pollen concentrations. Moreover, we developed the LUR approach by exploring the contributions of different data sources (remote sensing and land use) and scales of explanatory variables.

Based on the results, we draw three main conclusions. First, it is possible to spatially predict the fine-scale variation of grass pollen concentrations across an urban area using the LUR approach. An extensive evaluation of the modeling results is highly important. Moreover, based on the visual exploration of pollen predictions (Figure 4), models should be extrapolated beyond the calibration environment with care. Second, a remote sensing vegetation variable (TC greenness) outperformed land use variables in our study setting. Remote sensing–based indices have several strengths, which highlight their use in modeling and predicting pollen concentrations in human-modified environments. Third, statistically based predictive pollen models could probably be used in retrospective exposure assessment, if residential histories are available and pollen concentrations have been monitored or modeled for the corresponding period. The developed LUR approach demonstrated the possibility of predicting the spatial variability of mean pollen concentrations at breathing-zone level, contrary to urban background or regional scale usually pursued by the existing monitoring and modeling tools.

In the future, exposure assessment studies should not be based solely on (few) roof-level pollen monitoring sites due to the significant intra-urban variation of allergenic pollen concentrations. Instead, it would be highly valuable to combine both local- and regional-scale observations in studying spatially and temporally the relations between environmental determinants and concentrations of allergenic pollen. Moreover, hybrid modeling should be examined that combines physically (i.e., deterministic dispersion modeling) and statistically based models.

## Supplemental Material

(292 KB) PDF
