# Supplementary material for: Fine-Scale Exposure to Allergenic Pollen in the Urban Environment: Evaluation of Land Use Regression Approach
Source: Environ Health Perspect. 2015 Oct 9;124(5):619–26. doi: 10.1289/ehp.1509761 (PMC4858385; doi:10.1289/ehp.1509761)
Supplement: (292 KB) PDF [file ehp.1509761.s001.acco.pdf]

Environ Health Perspect
DOI: 10.1289/ehp.1509761

Note to readers with disabilities: *EHP* strives to ensure that all journal content is accessible to all readers. However, some figures and Supplemental Material published in *EHP* articles may not conform to 508 standards due to the complexity of the information being presented. If you need assistance accessing journal content, please contact ehp508@niehs.nih.gov. Our staff will work with you to assess and meet your accessibility needs within 3 working days.

Supplemental Material

Fine-Scale Exposure to Allergenic Pollen in the Urban Environment: Evaluation of Land Use Regression Approach

Jan Hjort, Timo T. Hugg, Harri Antikainen, Jarmo Rusanen, Mikhail Sofiev, Jaakko Kukkonen, Maritta S. Jaakkola, and Jouni J.K. Jaakkola

Table of Contents

Data and method descriptions

Sampling of grass pollen data

Hierarchical partitioning

Table S1. Weather conditions of the study period (8:00 am–5:00 pm; June 27th–July 21st, 2013). The whole sampling period was divided into two periods for the statistical analyses (period 1 = June 27–July 9 and period 2 = July 10–21) (see Data collection section for further details).

Table S2. Spearman's rank order correlation coefficient between the grass concentration (grains m^{-3}) and environmental variables for the determination of optimum buffer (all sample sites, n = 16).

Table S3. Spearman's rank order correlation coefficient between the grass concentration (grains m^{-3}) and environmental variables for the determination of optimum buffer size (two sites with extreme values were not included, n = 14).

Figure S1. Results of the hierarchical partitioning analysis for the determination of relative independent contribution (%) of environmental determinants. n = 16 refers to the data set including all sample sites and n = 14 to the data set, where two sites with extreme values

were excluded from the statistical analysis (see the Methods section). For the description of the environmental determinants, see the Methods section and Table 1.

References

Data and method descriptions

Sampling of grass pollen data

Rotorod-type samplers were used for pollen monitoring (Rantio-Lehtimäki et al. 1992). The samplers were equipped with a U-shaped metal rod (1.7 mm in diameter) and a power source (battery: NX, Powerfit S312/1.2S, Part No: AMP9033). Transparent Melinex tape coated with an adhesive (Vaseline; http://www.polleninfo.org) was fixed to the upper ends of the rods. The speed of rotation of each sampler was monitored with a Shimpo Instruments (Itasca) Shimpo DT-201 digital tachometer to ensure correct performance. The average speed of rotation of the arms was 2,173 rpm, varying between 2,040 and 2,275 rpm.

Rotorod-type samplers were attached on sampling poles to the height of 1.5 meters. To minimize problems with oversampling (Sterling and Lewis 1998) sampling periods were restricted to 30 min. The sampling in each site was started on the hour and was conducted in each sampling site twice a day in the morning between 8:00 and 11:30 and in the afternoon between 13:00 and 16:30. The chosen time frame as a whole corresponded the best diurnal pollination period of grasses. The sampling progressed in time and was conducted within lines as a cycle of four days in order to control temporal variation in pollen concentrations. The first sampling site starting at 8:00 or 13:00 in the first day was the last (fourth) site starting at 11:00 or 16:00 in the second day, the third (starting time 10:00/15:00) in the third day and the second (starting time 9:00/14:00) in the fourth day. On the fifth day, rotation started from the beginning. Other sampling sites followed the same rotation pattern in relation to time. The daily rotation occurred within morning and afternoon hours. Order of progress was identical in the morning and afternoon sessions. The sampling in the lines of Helsinki and Espoo was conducted on alternate days (Fig. 1).

After each 30-min period sampling tapes (total sample n = 914) were detached and mounted on slides with Gelvatol under a cover glass. Before analysis samples were stored at room

temperature in dust-proof preservation sheets for microscope slides. Pollen concentrations were determined by systematic sampling of microscopic fields and counted from the tapes under 400 x magnification with an optical microscope (Olympus BX43, Olympus Corporation). A single transect line from the middle of the sample, comprising 32% of the total sampling area, was examined. Pollen measurements were converted into volumetric equivalents expressed as the concentration of pollen grains per cubic meter of air sampled (grains m^{-3}; Raynor 1972).

Hierarchical partitioning

The hierarchical partitioning (HP) method is developed to overcome multicollinearity problems by using a mathematical hierarchical theorem by which the explanatory capacities of a set of variables can be estimated (Chevan and Sutherland 1991). Hierarchical partitioning (HP) employs goodness-of-fit measures for each of the 2^k potential models for k environmental determinants. In HP, the variances are partitioned so that the overall independent contribution of a given environmental variable can be computed (Chevan and Sutherland 1991). For example, the independent contribution of the urban land use variable is estimated by comparing goodness-of-fit measures for all models including the urban land use variable with their reduced version (i.e. the exact same model but without the urban land use variable) within each hierarchical level [e.g. with three environmental variables (2^3) there are eight possible models at four hierarchical levels (models with only intercept, one, two and three variables)]. The average improvement in fit for each hierarchical level that contains urban land use is then averaged across all hierarchies, giving the independent contribution of the urban land use variable. Thus, HP permits one to discriminate between those environmental determinants whose independent correlation with a response variable may be important from determinants that have little independent influence on the target feature (Chevan and Sutherland 1991). In this study, HP was conducted using 'hier.part' package

version 1.0-4 in statistical software R version 2.11.1 (http://r-project.org). To test the statistical significances of the environmental determinants a randomization test was performed (see Walsh and Mac Nally 2014). For both the considered datasets (n = 16 and n = 14 data set), a total of 256 regression models were generated to reveal the most meaningful environmental determinants for characterizing the variability of the whole-season grass pollen concentrations.

Table S1. Weather conditions of the study period (8:00 am–5:00 pm; June 27th–July 21st, 2013). The whole sampling period was divided into two periods for the statistical analyses (period 1 = June 27–July 9 and period 2 = July 10–21) (see Data collection section for further details).

Weather parameter[a]	Whole period, mean (min–max)	Period 1, mean (min–max)	Period 2, mean (min–max)
Air temperature at 2 m height	19.8°C (12.9–25.5 °C)	20.3°C (15.3–25.5°C)	19.4°C (12.9–24.6°C)
Wind speed (10 min average)	3.7 m/s (0.9–8.8 m/s)	3.4 m/s (0.9–6.7 m/s)	4.1 m/s (1.0–8.8 m/s)
Dominant wind direction	SW (41%)	SW (48%)	SW (32%)
Relative humidity of air	63.8% (33–98%)	67.3% (33–98%)	60.1% (34–96%)
Number of rainy 10-min periods (> 0.1 mm)	26	15	11

[a]Measured at Kaisaniemi meteorological station (Helsinki) at 10-min intervals (N60.18, E24.94, 4 m above sea level)

Table S2. Spearman's rank order correlation coefficient between the grass concentration (grains m^{-3}) and environmental variables for the determination of optimum buffer (all sample sites, n = 16).

Buffer size (m)	TC Brightness	TC Greenness	Wasteland	Fields	Parks	Deciduous forest	Mixed forest	Urban land use
25	0.514*	0.778***	0.576*		0.048		0.243	−0.532*
50	0.536*	0.792***●	0.576*		0.119●	0.571*	0.445	−0.713**
100	0.574*●	0.761***	0.622*		−0.007	0.545*	0.477●	−0.713**
300	0.297	0.736***	0.731***●	0.238	−0.120	0.637**●	0.239	−0.720**●
500	0.281	0.730***	0.725***	0.550*	−0.057	0.607*	0.189	−0.716**
1000	0.242	0.677**	0.633**	0.666**●	−0.265	0.536*	−0.205	−0.689**

The highest correlations with expected sign are shown by a symbol (●). For the description of the environmental variables, see the Methods section and Table 1.

Statistical significance: *$p < 0.05$, **$p < 0.01$, ***$p < 0.001$.

Table S3. Spearman's rank order correlation coefficient between the grass concentration (grains m^{-3}) and environmental variables for the determination of optimum buffer size (two sites with extreme values were not included, n = 14).

Buffer size (m)	TC Brightness	TC Greenness	Wasteland	Fields	Parks	Deciduous forest	Mixed forest	Urban land use
25	0.410	0.667**			0.187●		0.410	−0.365
50	0.443	0.689**●			0.177		0.615*●	−0.584*
100	0.491●	0.643*	0.310		0.073	0.172	0.476	−0.584*
300	0.119	0.619*	0.594*	0.076	−0.122	0.492●	0.236	−0.595*
500	0.095	0.610*	0.615*●	0.375	−0.053	0.418	0.161	−0.588*
1000	0.026	0.575*	0.470	0.499●	−0.128	0.381	0.042	−0.619*●

The highest correlations are shown by a symbol (●). For the description of the environmental variables, see the Methods section and Table 1.

Statistical significance: *$p < 0.05$, **$p < 0.01$, ***$p < 0.001$.

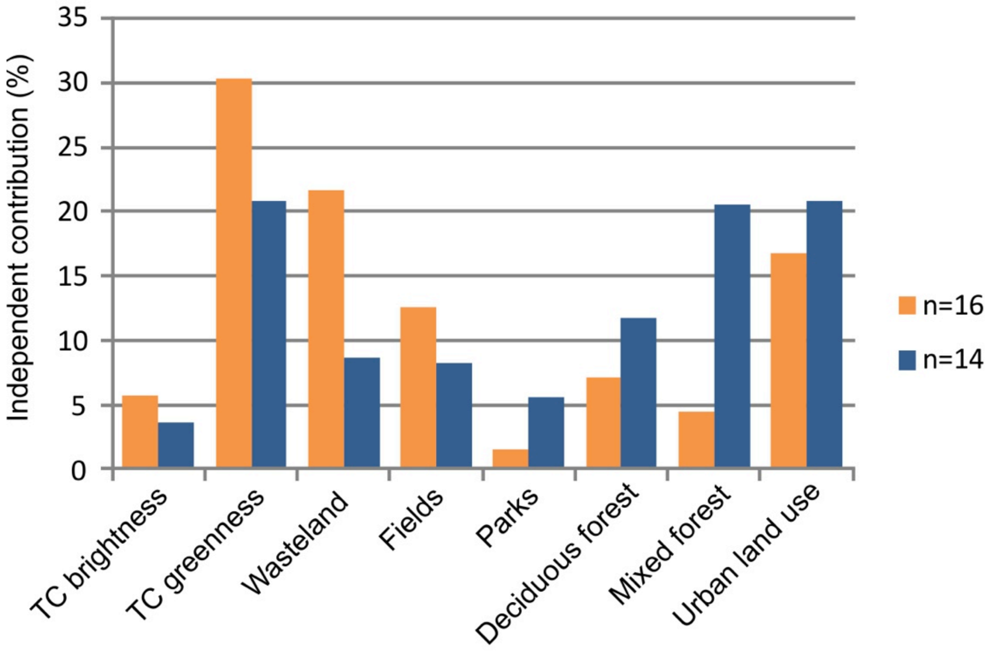

Figure S1. Results of the hierarchical partitioning analysis for the determination of relative independent contribution (%) of environmental determinants. n = 16 refers to the data set including all sample sites and n = 14 to the data set, where two sites with extreme values were excluded from the statistical analysis (see the Methods section). For the description of the environmental determinants, see the Methods section and Table 1.

References

Chevan A, Sutherland M. 1991. Hierarchical partitioning. Am Stat 45:90–96.

Rantio-Lehtimäki A, Helander ML, Karhu K. 1992. Does cutting of mugwort stands affect airborne pollen concentrations? Allergy 47:388–390.

Raynor GS. 1972. Sampling particulates with rotating arm impaction samplers. In: Ecological System Approaches to Aerobiology. I. Identification of Component Elements and Their Functional Relationships, International Biological Program, Aerobiology Program, US/ IBP Aerobiology Program Handbook N.2 (Benninghoff WS, Edmonds RL, eds). Ann Harbor, MI:The University of Michigan, 82–105.

Sterling DA, Lewis RD. 1998. Pollen and fungal spores indoor and outdoor of mobile homes. Ann Allergy Asthma Immunol 80:279–285.

Walsh C, Mac Nally R. 2014. Package ‘hier.part’. Version 1.0-4. Available: http://cran.r-project.org/web/packages/hier.part/hier.part.pdf [accessed 20 January 2015].
